# Large-Scale Analysis of Determinants, Stability, and Heritability of High-Density Lipoprotein Cholesterol Efflux Capacity

**DOI:** 10.1161/ATVBAHA.117.309201

**Published:** 2017-09-27

**Authors:** Andrea L. Koekemoer, Veryan Codd, Nicholas G.D. Masca, Christopher P. Nelson, Muntaser D. Musameh, Bernhard M. Kaess, Christian Hengstenberg, Daniel J. Rader, Nilesh J. Samani

**Affiliations:** From the Department of Cardiovascular Sciences and NIHR Leicester Biomedical Centre, University of Leicester, United Kingdom (A.L.K., V.C., N.G.D.M., C.P.N., M.D.M., N.J.S.); German Heart Center, Technische Universität, Munich, Germany (B.M.K., C.H.); Department for Internal Medicine I, St. Josefs-Hospital, Wiesbaden, Germany (B.M.K.); German Center for Cardiovascular Research, Partner Site Munich Heart Alliance, Munich, Germany (C.H.); and Department of Genetics, Perelman School of Medicine, University of Pennsylvania, Philadelphia (D.J.R.).

**Keywords:** genetics, lipoprotein(a), phospholipids, triglycerides, ultracentrifugation

## Abstract

**Objective—:**

Cholesterol efflux capacity (CEC) has emerged as a biomarker of coronary artery disease risk beyond plasma high-density lipoprotein (HDL) cholesterol (HDL-C) level. However, the determinants of CEC are incompletely characterized. We undertook a large-scale family-based population study to identify clinical, biochemical, and HDL particle parameter determinants of CEC, characterize reasons for the discordancy with HDL-C, quantify its heritability, and assess its stability over 10 to 12 years.

**Approaches and Results—:**

CEC was quantified in 1988 individuals from the GRAPHIC (Genetic Regulation of Arterial Pressure of Humans in the Community) cohort, comprising individuals from 2 generations from 520 white nuclear families. Serum lipid and lipoprotein levels were determined by ultracentrifugation or nuclear magnetic resonance and HDL particle size and number quantified by nuclear magnetic resonance. Ninety unrelated individuals had repeat CEC measurements in samples collected after 10 to 12 years. CEC was positively correlated with HDL-C (*R*=0.62; *P*<0.0001). Among clinical and biochemical parameters, age, systolic blood pressure, alcohol consumption, serum albumin, triglycerides, phospholipids, and lipoprotein(a) were independently associated with CEC. Among HDL particle parameters, HDL particle number, particle size, and apolipoprotein A-II level were independently associated with CEC. Serum triglyceride level partially explained discordancy between CEC and HDL-C. CEC measurements in samples collected 10 to 12 years apart were strongly correlated (*r*=0.73; *P*<0.0001). Heritability of CEC was 0.31 (*P*=3.89×10^−14^) without adjustment for HDL-C and 0.13 (*P*=1.44×10^−3^) with adjustment.

**Conclusions—:**

CEC is a stable trait over time, is influenced by specific clinical, serum, and HDL particle parameters factors beyond HDL-C, can be maintained in persons with a low plasma HDL-C by elevated serum triglyceride level, and is modestly independently heritable.

## Abstract 2

Supplemental Digital Content is available in the text.

Numerous studies have shown that low levels of plasma high-density lipoprotein cholesterol (HDL-C) are strongly associated with an increase in the risk of coronary artery disease.^1^ However, several therapies that increase plasma HDL-C have not shown the anticipated clinical benefit based on the epidemiological association.^2–4^ In addition, genetic factors that raise HDL-C are not associated with reduced coronary artery disease.^5,6^ HDL, however, is a heterogeneous population of particles that differ in size and composition,^7^ of which HDL-C is a crude measure. Furthermore, HDL has a variety of functions, the best established being its ability to promote the efflux of cholesterol from lipid-laden macrophages for ultimate return to the liver and biliary excretion (reverse cholesterol transport).^7,8^ HDL subfractions differ widely in their ability to remove cholesterol from macrophages (cholesterol efflux capacity [CEC]).^9,10^ Hence, HDL functionality, in particular CEC, could be a better disease predictor than total HDL.

A method has been developed of quantifying CEC^11^ and used to explore relationships with HDL-C and cardiovascular disease.^12–14^ HDL-C concentration is a modest predictor of CEC,^11–14^ and individuals with similar HDL-C levels can have very different CEC.^11^ A strong body of data indicates that CEC is inversely associated with atherosclerotic cardiovascular disease even after adjusting for HDL-C and apolipoprotein AI levels. CEC is negatively correlated with carotid intima-media thickness and angiographic coronary artery disease.^12^ Moreover, CEC is inversely associated with the incident cardiovascular events, independently of HDL-C level.^13,14^ CEC as a measure of HDL function may therefore be a better marker of disease risk that may be more likely to be causally related to disease than is HDL-C concentration. Indeed, there has been substantial interest in therapeutic interventions that increase CEC rather than HDL-C per se.^15^

Despite the potential importance of CEC as a predictor of risk and a target for therapeutic intervention, determinants of CEC have not been fully characterized. Here, we sought to identify determinants of CEC, including clinical and biochemical characteristics as well as features of the HDL particle, all assessed in the same individuals. We also sought to identify factors that may explain discordancy between HDL-C level and CEC and determined the stability and heritability of CEC.

## Materials and Methods

Materials and Methods are available in the online-only Data Supplement. Analyses were performed in the GRAPHIC (Genetic Regulation of Arterial Pressure of Humans in the Community) cohort,^16^ comprising individuals from 520 white nuclear families of European descent.

## Results

### Characteristics of the Study Population

The clinical and biological characteristics of the 1932 individuals studied are shown in Table 1. The mean age was 39.4±14.5 years (range, 18–61 years), and 49% were male. The mean relative CEC was 1.05±0.16 (range, 0.35–1.70). The distribution of CEC is shown in Figure I in the online-only Data Supplement.

**Table 1.**
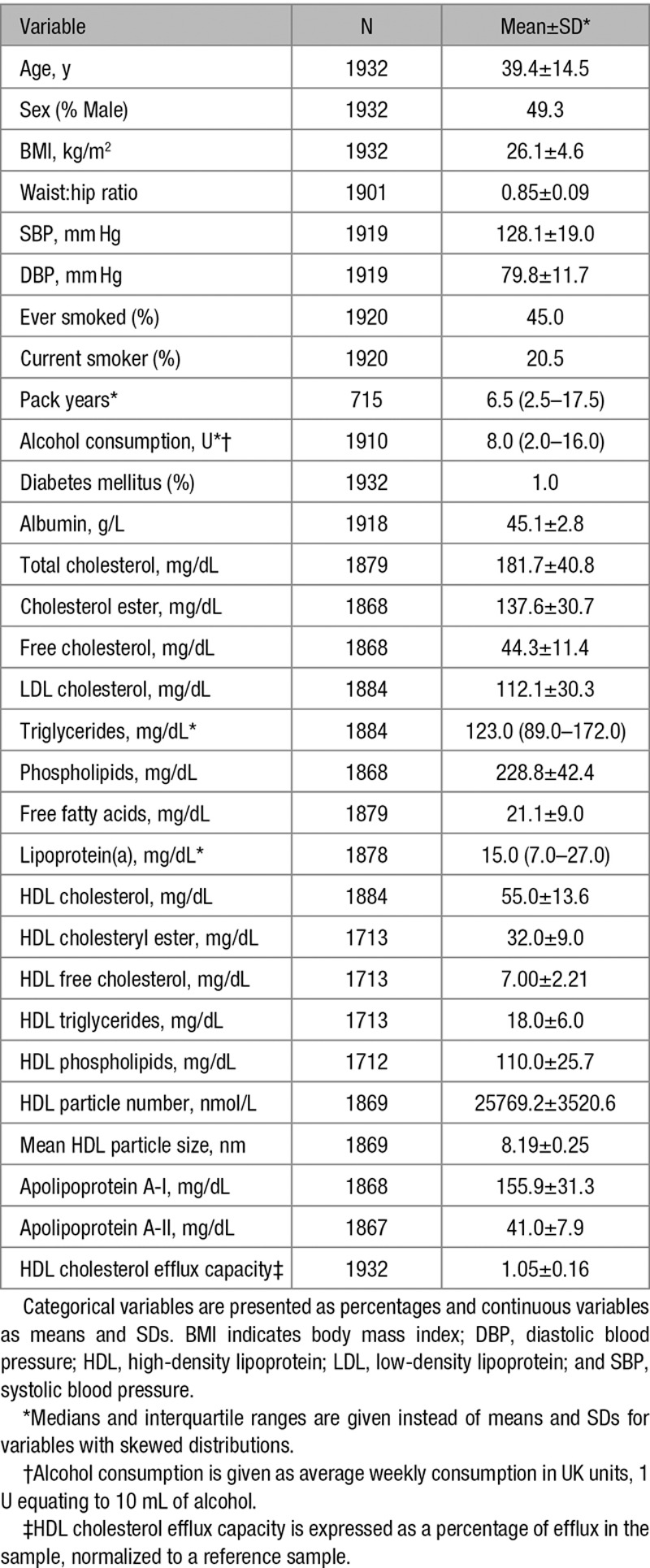
Characteristics of the GRAPHIC (Genetic Regulation of Arterial Pressure of Humans in the Community) Cohort

### Relationships Between CEC and Clinical and Serum Parameters

As previously reported, CEC positively correlated with plasma HDL-C level (*R*=0.62; *P*<0.0001; Figure II in the online-only Data Supplement). Univariate analyses of the relationship between CEC and clinical and serum parameters before and after adjustment for plasma HDL-C are shown in Table 2. Several factors, both clinical and biochemical, showed highly significant associations with CEC even after adjustment for plasma HDL-C (although it should be noted that in terms of effect size, these associations were generally rather small). Interestingly, neither current smoking nor diabetes mellitus status associated with CEC. After adjustment for plasma HDL-C, women showed a borderline lower CEC compared with men.

**Table 2.**
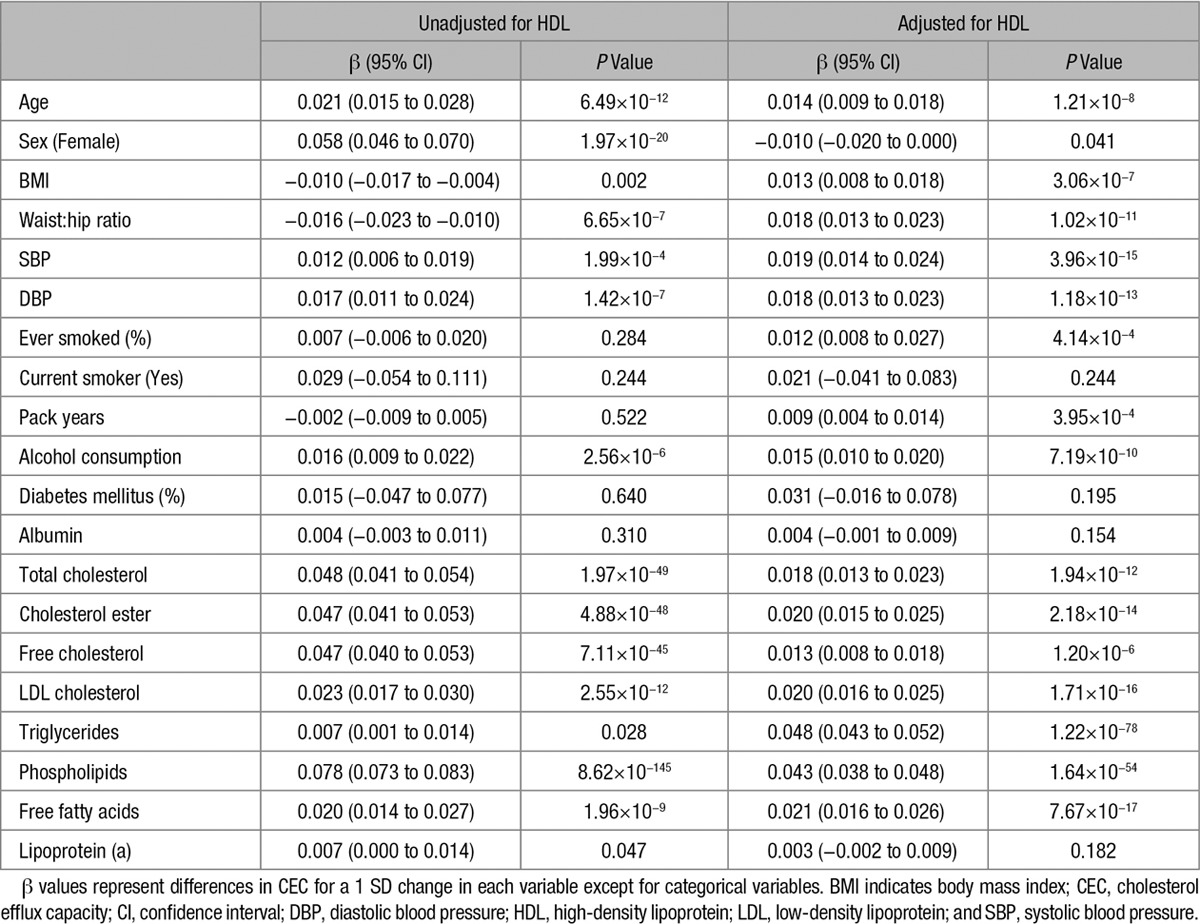
Relationship Between Clinical and Serum Parameters and CEC

In a multivariate model adjusted for plasma HDL-C and based on 1669 individuals with full data on all parameters included (Materials and Methods), 7 factors independently associated with CEC at *P*<0.05—age, systolic blood pressure, alcohol consumption, and serum levels of albumin, triglycerides, phospholipids, and lipoprotein(a) (Lp(a)) (Table 3) with no evidence of multicollinearity in the final model.

**Table 3.**
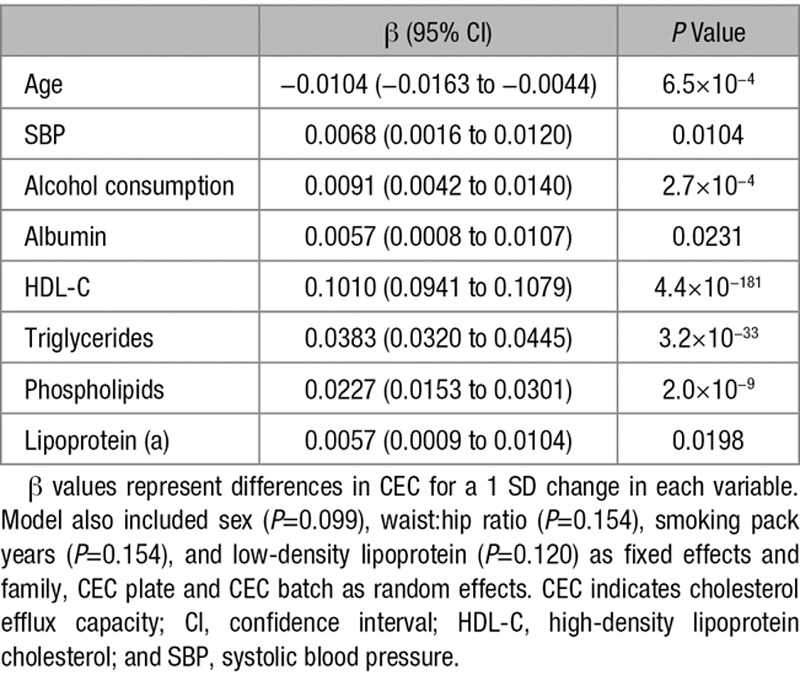
Clinical and Serum Parameters Showing Independent Association Within CEC

### HDL Particle Parameters and CEC

We next investigated the relationship between other features of HDL particles and CEC. Several of these parameters showed an association with CEC even after adjustment for plasma HDL-C (Table 4). Specifically, there was a positive correlation between CEC and HDL particle number (*R*=0.45; *P*<0.0001) and with HDL particle size (*R*=0.49; *P*<0.0001; Figure II in the online-only Data Supplement).

**Table 4.**
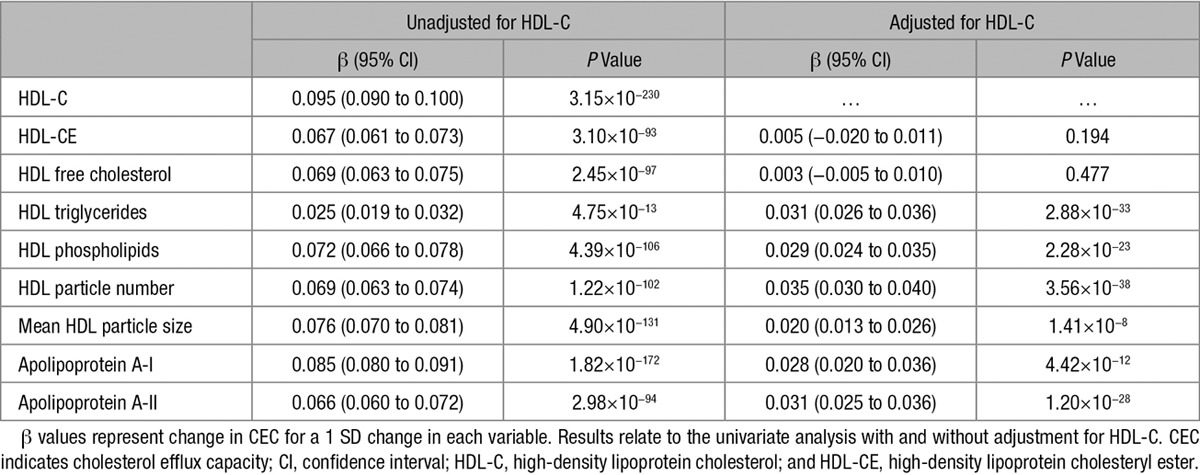
Relationship Between HDL Particle Parameters and CEC

In a multivariate model, adjusting for plasma HDL-C and the clinical and serum parameters associated with efflux, 3 HDL particle parameters remained independently associated with CEC: HDL particle number (β=0.018; 95% confidence interval [CI], 0.010–0.025; *P*<0.0001), HDL particle size (β=0.022; 95% CI, 0.013–0.032; *P*<0.0001), and apolipoprotein A-II level (β=0.008; 95% CI, 0.001–0.015; *P*=0.032).

### Variables That Explain Discordance Between CEC and Plasma HDL-C

Although CEC is overall significantly associated with plasma HDL-C, some individuals exhibited discordance between CEC and plasma HDL-C (ie, a high CEC despite low plasma HDL-C or a low CEC despite high plasma HDL-C; Figure II in the online-only Data Supplement). We therefore explored whether any clinical, serum, or HDL particle parameters explained this discordance. For this analysis, each individual was categorized into low (bottom tertile), normal (middle tertile), or high (upper tertile) for both CEC and HDL-C. From cross-tabulation, individuals with concordance (high CEC and high HDL-C levels; low CEC and low HDL-C levels) and discordance (low CEC and high HDL-C levels; high CEC and low HDL-C levels) were identified. The clinical and biological characteristics of the 4 groups are shown in Table I in the online-only Data Supplement. After adjusting for HDL-C, several variables influenced the odds of an individual being in the low CEC/high HDL-C group or in the high CEC/low HDL-C group (Table 5; Table II in the online-only Data Supplement). Adjusting for the number of tests undertaken plasma triglyceride level was the only factor that was significantly associated with partitioning individuals in both directions. Individuals with higher plasma triglyceride levels were less likely to be in the low CEC/high HDL-C group (odds ratio, 0.20; 95% CI, 0.08–0.51 per 1 SD higher plasma triglyceride) and more likely to be in the high CEC/low HDL-C group (odds ratio, 2.03; 95% CI, 1.55–2.65 per 1 SD higher plasma triglyceride) compared with the concordant groups (Table 5).

**Table 5.**
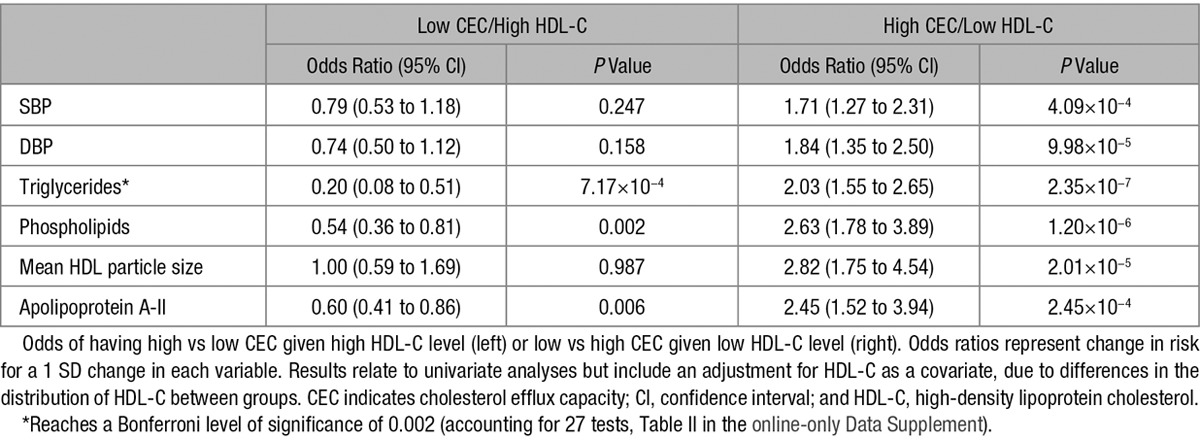
Variables Associated With Either a Low CEC and High Plasma HDL-C Level or a High CEC With a Low Plasma HDL-C Level

### Stability of CEC

CEC measured ≈2 to 3 years apart using the same serum sample from 90 individuals showed high correlation (*r*=0.81; *P*<0.0001; Figure [A]). CEC measurements in different serum samples collected 10 to 12 years apart from the same individual were also strongly correlated (*r*=0.73; *P*<0.0001; Figure [B]). The average CEC values in these individuals (relative to the pooled sample) were also remarkably similar between the baseline samples and the samples taken 10 to 12 years later (CEC at baseline=0.99±0.17, CEC at follow-up=0.98±0.13; *P*=0.658). HDL-C measured in the same samples (n=82) also showed a high degree of correlation (*r*=0.82; *P*<0.0001) (Figure [C]).

**Figure.**
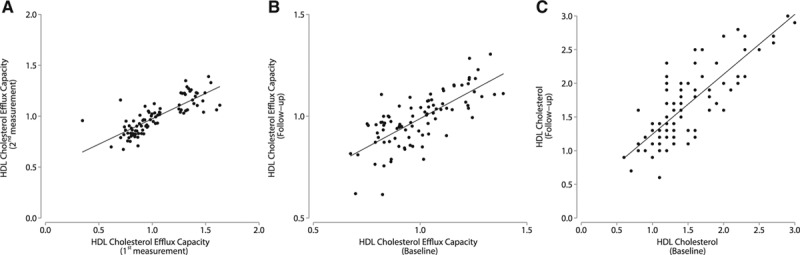
**A**, Correlation of duplicate measurements of cholesterol efflux capacity (CEC) performed in individuals (n=90) using the same serum sample measured 2 to 3 years apart. **B**, Correlation of CEC in individuals (n=90) on serum obtained at baseline and at 10- to 12-year follow-up. **C**, Correlation of high-density lipoprotein cholesterol (HDL-C) in individuals (n=82) in serum obtained at baseline and at 10- to 12-year follow-up.

### Heritability of CEC and Exploratory Association Analysis of Selected Genetic Variants With CEC

Narrow sense heritability estimate (*h*^2^) for plasma HDL-C accounting for age, sex, batch, and plate was 0.51 (*P*=3.08×10^−33^). A similar analysis for cholesterol CEC gave values of 0.31 (*P*=3.89×10^−14^) without adjustment for HDL-C and 0.13 (*P*=1.44×10^−3^) after adjusting for plasma HDL-C.

Of the 55 single nucleotide polymorphisms associated with HDL-C identified through large-scale genome-wide association studies^17^ that we tested, 7 variants (most notably lead variants at *LIPC, CETP* [cholesteryl ester transfer protein], and *LIPG*) showed a nominal association (*P*<0.05) with HDL-C in the GRAPHIC parental generation and 7 variants with CEC unadjusted for HDL-C (most notably variants at *LIPC* and *ANGPTL8* [angiopoeitin-like protein]; Table III in the online-only Data Supplement). Interestingly, the association of rs737337 at the *ANGPTL8* locus was more significant for CEC (*P*=4.4×10^−3^) than for HDL-C (*P*=0.088) and remained borderline significant (*P*=0.053) after adjusting for HDL-C (Table III in the online-only Data Supplement).

## Discussion

We report the first family-based study of CEC and the most detailed study to date of the determinants of CEC beyond plasma HDL-C level, including an assessment of its stability over a decade and an estimate of its heritability. Although previous studies have examined the association of CEC with a variety of clinical, biochemical, and HDL particle parameters,^11–14^ to the best of our knowledge, ours is the first study to examine the relationship between all of these parameters and CEC in the same subjects.

As expected, CEC was positively correlated with plasma HDL-C level with an *r*=0.62. This is slightly higher than has been reported in previous studies^11–14^ but nonetheless confirms that CEC is not simply a surrogate for plasma HDL-C level. We also observed a moderate independent correlation between CEC and both HDL particle number and HDL particle size. However, even these 3 parameters (plasma HDL-C, HDL particle number, and HDL particle size) do not fully explain CEC, suggesting that other factors are involved in determining the CEC.

Several clinical characteristics showed univariate association with CEC, even after adjustment for HDL-C, including age, sex, blood pressure, body mass index, waist/hip ratio smoking history, and alcohol consumption, although, interestingly, not diabetes mellitus (Table 2). Many of these correlations have been reported previously.^11–14^ However, the majority of these associations with clinical characteristics became nonsignificant in a multivariate model that included biochemical parameters, suggesting that these associations are because of a primary association of the clinical characteristics with biochemical parameters which affect CEC. The exceptions were age, systolic blood pressure, and alcohol consumption (Table 3). Interestingly, in the univariate analysis, there was a positive association of age with CEC, whereas in the multivariate model the association was inverse suggesting that the independent effect of age on CEC is a decline with increasing age. The reason for the association of CEC with systolic blood pressure is unclear but a possible reason for the association with alcohol consumption is a direct effect of alcohol intake on ABCA1 (ATP-binding cassette transporter 1) expression.^18,19^

Several plasma lipid parameters—triglycerides, phospholipids and Lp(a)—were positively associated with CEC (Table 3). These results are supported by other published findings, in particular, implicating a role of phospholipids in cholesterol efflux.^20–22^ The association with Lp(a) is also biologically plausible as in vitro studies have shown that Lp(a) upregulates ABCA1 in liver cells via scavenger receptor B-1.^23^ Interestingly, the association of CEC with triglycerides and Lp(a) is in a direction associated with increased risk of coronary artery disease, suggesting that the overall relationship of CEC to cardiovascular risk may involve a balance of different influences affecting CEC. In univariate, there was no significant relationship between CEC and serum albumin level (Table 2); however, in the multivariate model, albumin level was positively associated with CEC (Table 3). It has been reported that albumin can serve as a cholesterol acceptor and promote cholesterol efflux,^24,25^ and this may explain the observed association.

HDL particle number, HDL particle size, and apolipoprotein AII were the main components of HDL that influenced CEC. Rohatgi et al^13^ also reported that HDL particle size was positively correlated with CEC, although in their study the association was not significant. Previous studies have also shown a positive correlation between CEC and apolipoprotein AII levels which may be attributed to the pre-β HDLs containing apolipoprotein AII.^12,26^

The reasons for the discordancy between CEC and plasma HDL-C level remain poorly understood. Here, we show that one explanation relates to serum triglyceride levels. In a discordance analysis, we observed that individuals with high serum triglycerides were more likely to have a high CEC even if HDL-C levels were low and vice versa. Our finding is consistent with a recent study that reported increased CEC in individuals with high triglyceride and low HDL-C levels.^27^ Furthermore, previous findings demonstrated that sera from individuals with high triglyceride levels but low HDL-C levels exhibited increased ABCA1-mediated efflux because of increased pre-β HDL levels.^28,29^ This suggests that although there is an overall inverse relationship between plasma triglyceride and HDL-C, the positive association between serum triglyceride level and CEC contributes to the variability observed between plasma HDL-C and CEC. Further, our findings suggest that an effect on CEC is not a major mechanism contributing to the emerging causal role of triglycerides in atherosclerosis.^30^

The family-based nature of GRAPHIC allowed us to explore the heritability of CEC. Our analysis showed that this was weaker than for plasma HDL-C but remained significant after adjustment for plasma HDL-C, suggesting that specific genetic variants may affect CEC. In an exploratory analysis to identify such variants, we examined the association with CEC (and HDL-C) of variants associated with HDL-C identified through large-scale genome-wide association studies^17^ to see if any of these had a stronger association with CEC. Fully recognizing the underpowered nature of the analysis, which meant that only a few variants showed even a nominal association with HDL-C in our study, we observed that the association of the lead variant (rs737337) at the *ANGPTL8* locus was more significant for CEC than for HDL-C and remained borderline significant (*P*=0.053) after adjusting for HDL-C (Table III in the online-only Data Supplement). *ANGPTL8* codes for a secreted protein, angiopoietin-like protein 8, which with other members of the ANGPTL family negatively regulates lipoprotein lipase and increases plasma triglyceride level.^31^ Given the influence of trigycerides on CEC, as discussed earlier, an effect though triglycerides could provide a mechanism by which variants at this locus affects CEC. However, the association first needs to be confirmed in much larger studies, but our preliminary analysis suggests that specific variants that specifically affect CEC may be tractable.

Several further limitations of our study need to be acknowledged. Individuals included in the study are all of European ancestry and come from a small geographical location so may not fully represent the wider population or other ethnic groups. Samples were stored at −80°C before analysis without thawing to preserve sample integrity. However, the storage time between the lipid/lipoprotein and the CEC measurements did vary (≈4 versus ≈8 years, respectively). Although there may be a modest effect of this on the values obtained, we feel it is likely to be small given the good concordance between baseline samples that were measured 3 years apart that had also undergone an additional freeze and thaw cycle. Although we observed statistically significant associations of CEC with several clinical, biochemical, and HDL particle parameters beyond HDL-C, many of the associations were small and their clinical relevance remains to be determined. Finally, given the relatively young age of the GRAPHIC cohort and the relatively short duration of follow-up, relatively few cardiovascular events have occurred to relate CEC measurements to outcomes.

In summary, we have performed a large family-based study of the determinants of CEC. Our results establish that CEC is a stable trait over time, is influenced by specific clinical, serum, and HDL particle parameters factors beyond HDL-C, can be maintained in persons with a low plasma HDL-C by elevated serum triglyceride level, and is modestly independently heritable.

## Acknowledgments

We are grateful to Dr Anatol Kontush, PhD, for his comments on the article.

## Sources of Funding

The GRAPHIC study (Genetic Regulation of Arterial Pressure of Humans in the Community) was funded by the British Heart Foundation (BHF). Drs Codd, Nelson, and Samani are funded by the BHF, and Dr Samani is a National Institute for Health Research Senior Investigator.

## Disclosures

None.

## Supplementary Material

## Notes

Highlights

- Cholesterol efflux capacity is a stable trait that is influenced by number of clinical, biochemical, and high-density lipoprotein particle features, beyond high-density lipoprotein cholesterol.
- Triglyceride levels partly explain discordancy between cholesterol efflux capacity and high-density lipoprotein cholesterol.
- Cholesterol efflux capacity has a modest heritability independently of high-density lipoprotein cholesterol.
