# Supplementary material for: Large-Scale Analysis of Determinants, Stability, and Heritability of High-Density Lipoprotein Cholesterol Efflux Capacity
Source: Arterioscler Thromb Vasc Biol. 2017 Sep 27;37(10):1956–62. doi: 10.1161/ATVBAHA.117.309201 (PMC5627541; doi:10.1161/ATVBAHA.117.309201)
Supplement: Supplementary file 1 [file atv-37-1956-s001.pdf]

Material and Methods

Study population

Analyses were carried out in the GRAPHIC (Genetic Regulation of Arterial Pressure of Humans in the Community) cohort.[1] The GRAPHIC study comprises individuals from 520 white nuclear families of European descent recruited from the general population in Leicestershire, UK. Families were included if both parents aged 40-60 years and two offspring ≥18 years wished to participate. Families were recruited through participating family practitioners in Leicestershire, UK, between 2003 and 2005. The study was approved by the Leicestershire Research Ethics Committee (LREC Number 6463) and all participants provided written informed consent.

Sample storage

Serum was prepared, aliquoted and stored at -80C. Previously unthawed aliquots were used for lipid, lipoprotein and CEC measurements. For repeat assessment of CEC a second thaw of the aliquot was performed.

Lipid and lipoprotein measurements

Serum lipid and lipoprotein measurements were determined by ultracentrifugation at the Clinical Institute of Medical and Chemical Laboratory Diagnostics, Medical University of Graz, Graz, Austria except for HDL-C, low-density lipoprotein (LDL) cholesterol and triglycerides which were determined by nuclear magnetic resonance (NMR) spectroscopy. HDL particle size and number were assessed by NMR spectroscopy.[2,3]

CEC assay

CEC was quantified in 1988 individuals of the GRAPHIC cohort using a previous established method that quantifies the ability of an individual's serum to accept radiolabeled cholesterol from a mouse macrophage cell line (J744) *ex vivo*.[4] Briefly, J774 macrophage cells

(American Type Culture Collection) were plated and incubated with 2µCi/ml radiolabeled cholesterol ([^{3}H]-cholesterol, Perkin Elmer) overnight. The labelling media was removed and ABCA1 was up-regulated by a 24 hour incubation with 0.3 mM 8-(4-chlorophenylthio)-cyclic AMP (Sigma). After the up-regulation period, individual serum samples were depleted of apoB lipoproteins with polyethylene glycol (MW 8000; Sigma) and then diluted to 2% serum and added to cells for 4 hours. After 4 hours the amount of [^{3}H]-cholesterol that has been effluxed from the cells was measured by removing and filtering the media and liquid scintillation counting. All incubation steps were in the presence of 2µg/ml acylcoenzyme A:cholesterol acyltransferase inhibitor (Sigma) to block the breakdown of free cholesterol to cholesterol esters.

All CEC assays were performed in duplicate. Control wells not exposed to individual's serum were included to measure both [^{3}H]-cholesterol uptake by the cells and the amount of [^{3}H]-cholesterol removed to the serum free media by passive diffusion. Percent CEC was calculated for each sample by the following formula: [(radioactivity in medium – radioactivity in serum-free medium) ÷ radioactivity uptake by cells] × 100. Serum was collected from 20 healthy volunteers to identify individuals with high and low CEC and used as controls on each plate to allow for monitoring of plate to plate and day to day variations. A pooled serum control from 20 healthy volunteers was also included on each plate to allow for inter-assay variation and values for individual's serum were standardized to this. The mean inter-assay coefficient of variation calculated for the "high" and "low" controls was 4.7% and 6.3% respectively.

Stability of CEC

About half the GRAPHIC subjects attended for a follow-up assessment in 2014-2015, between 10-12 years after their first assessment. Fresh blood samples were collected from these individuals, using the same operating procedures as at the initial visit. This gave us an opportunity to examine the longitudinal stability of CEC. We selected 90 unrelated

individuals with CEC values across the range of measurements from the initial visit and re-measured their CEC again from an aliquot of serum from the initial visit as well as from the second visit. This allowed us to assess the stability of CEC in an individual over time as well as to assess the stability of CEC measurement in samples stored for a long period and measured twice with the two measurements between 2-3 years. In parallel, to assess the stability of HDL-C we measured HDL-C levels from samples collected at the initial and repeat visit in 82 subjects.

Statistical analysis

Linear mixed effects models were fitted to assess the relationships between CEC and sets of clinical, biochemical and HDL parameters. We accounted for the potential effects of batch, plate and correlation within families by including these terms as random effects within the mixed model. We also adjusted for plasma HDL-C in all models by including it as a covariate.

We performed univariate and multivariate analyses. In the multivariate analyses, we used a backwards model selection procedure, using the Akaike Information Criterion (AIC), removing variables from the mixed model where this resulted in a lower model AIC, and therefore a better fit to identify sets of variables that best determine CEC. The clinical variables included in the analyses were age, age^2 (because of the family structure of GRAPHIC), sex, body mass index (BMI), waist: hip ratio, systolic blood pressure, diastolic blood pressure, status with regard to ever smoked, current smoker, pack years, alcohol consumption, and presence or absence of diabetes. The biochemical variables included serum albumin level, cholesterol ester level, LDL cholesterol level, triglyceride level, phospholipid level, free fatty acid level, lipoprotein (a) level and HDL-C level. Data which were non-normally distributed (BMI, WHR, triglycerides, lipoprotein (a), alcohol consumption and smoking pack years) were transformed using rank-based inverse normalised transformations. All continuous variables were Z-standardised to aid interpretation of

coefficients. All statistical analyses were undertaken in R (http://www.R-project.org/) with models performed using the lme4 package (http://CRAN.R-project.org/package=lme4). To assess multicollinearity we calculated the variance inflation factors of the variables in the final model and considered any factor <3 to be acceptable. We tested the heteroskedasticity of the population level residuals using the Breusch-Pagan test[5].

Heritability was calculated using variance components analysis in Sequential Oligogenic Linkage Analysis Routines (SOLAR v7.2.5). Adjustments were made for age, sex, batch and plate.

In an exploratory analysis, to assess the association with CEC of selected genetic single nucleotide polymorphisms (SNPs) associated with HDL-C in genome wide-association studies,[6] we used two different models for CEC, each adjusting for age, sex, CEC plate and CEC batch, with the second model also adjusting for HDL-C. These analyses were carried out in subjects in the parental generation of GRAPHIC where we have genome-wide genotype data available.[7] For comparison, we also examined the association with HDL-C, adjusted for age and sex only. The sample sizes available for these analyses were 850 and 1,011 for CEC and HDL-C, respectively.

References

1. Tobin MD, Tomaszewski M, Braund PS, Hajat C, Raleigh SM, Palmer TM, Caulfield M, Burton PR, Samani NJ. Common variants in genes underlying monogenic hypertension and hypotension and blood pressure in the general population. Hypertension. 2008;51:1658-1664.

2. Kaess B, Fischer M, Baessler A, Stark K, Huber F, Kremer W, Kalbitzer HR, Schunkert H, Riegger G, Hengstenberg C. The lipoprotein subfraction profile: heritability and identification of quantitative trait loci. J Lipid Res. 2008;49:715-723.

3. Kaess BM, Tomaszewski M, Braund PS, Stark K, Rafelt S, Fischer M, Hardwick R, Nelson CP, Debiec R, Huber F, Kremer W, Kalbitzer HR, Rose LM, Chasman DI, Hopewell J, Clarke R, Burton PR, Tobin MD, Hengstenberg C & Samani NJ. Large-scale candidate gene analysis of HDL particle features. PLoS One. 2011;6:e14529.

4. de la Llera-Moya M, Drazul-Schrader D, Asztalos BF, Cuchel M, Rader DJ, Rothblat GH. The ability to promote efflux via ABCA1 determines the capacity of serum specimens with similar high-density lipoprotein cholesterol to remove cholesterol from macrophages. Arterioscler Thromb Vasc Biol. 2010;30:796-801.

5. Breusch TS, Pagan AR. A Simple Test for Heteroskedasticity and Random Coefficient Variation. Econometrica. 1979:47:1287-1294

6. Global Lipids Genetics Consortium. Discovery and Refinement of Loci Associated with Lipid Levels. Nat Genet. 2013;45:1274–1283.

7. Nelson CP, Schunkert H, Samani NJ, Erridge C. Genetic analysis of leukocyte type-I interferon production and risk of coronary artery disease. Arterioscler Thromb Vasc Biol. 2015:35:1456-1462.
