# Supplementary material for: Large-Scale Analysis of Determinants, Stability, and Heritability of High-Density Lipoprotein Cholesterol Efflux Capacity
Source: Arterioscler Thromb Vasc Biol. 2017 Sep 27;37(10):1956–62. doi: 10.1161/ATVBAHA.117.309201 (PMC5627541; doi:10.1161/ATVBAHA.117.309201)
Supplement: Supplementary file 2 [file atv-37-1956-s002.pdf]

ONLINE SUPPLEMENTAL MATERIAL

Large scale analysis of determinants, stability and heritability of HDL cholesterol efflux capacity

Koekemoer A et al.

Supplementary Figure I : Distribution of HDL CEC in the GRAPHIC cohort.

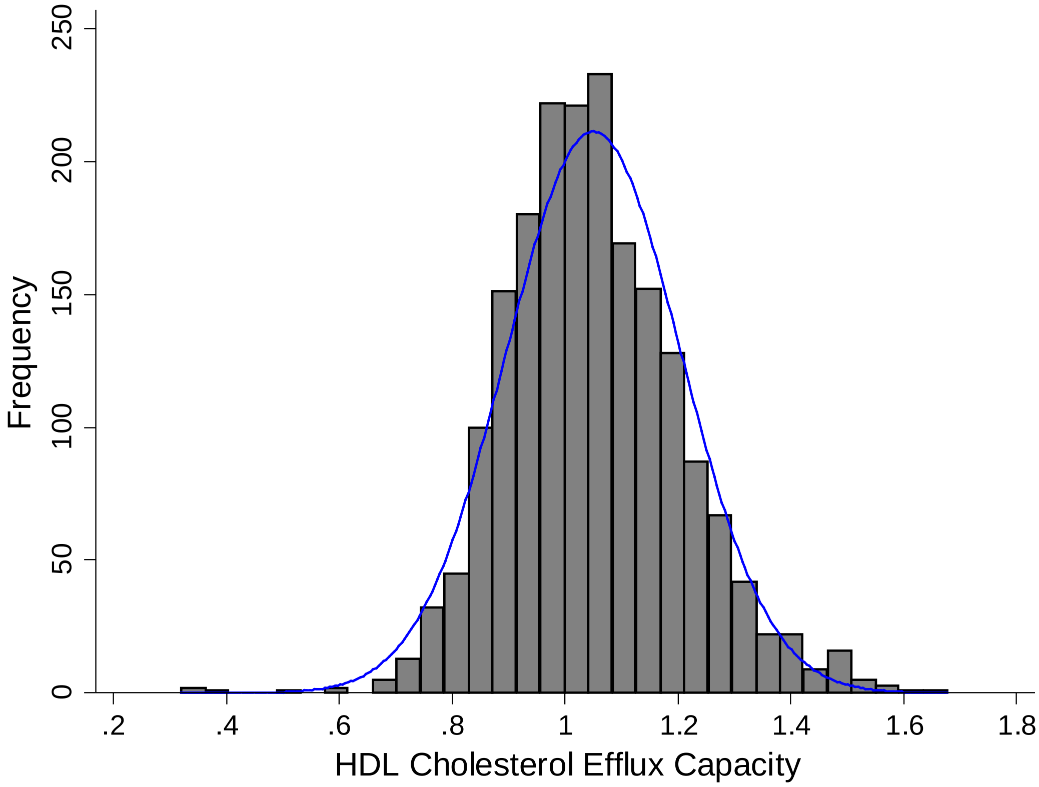

Histogram of CEC distribution in the GRAPHIC cohort; the solid blue line denotes the normal distribution.

Supplementary Figure II: Association between CEC and plasma HDL-C, HDL particle number and HDL particle size

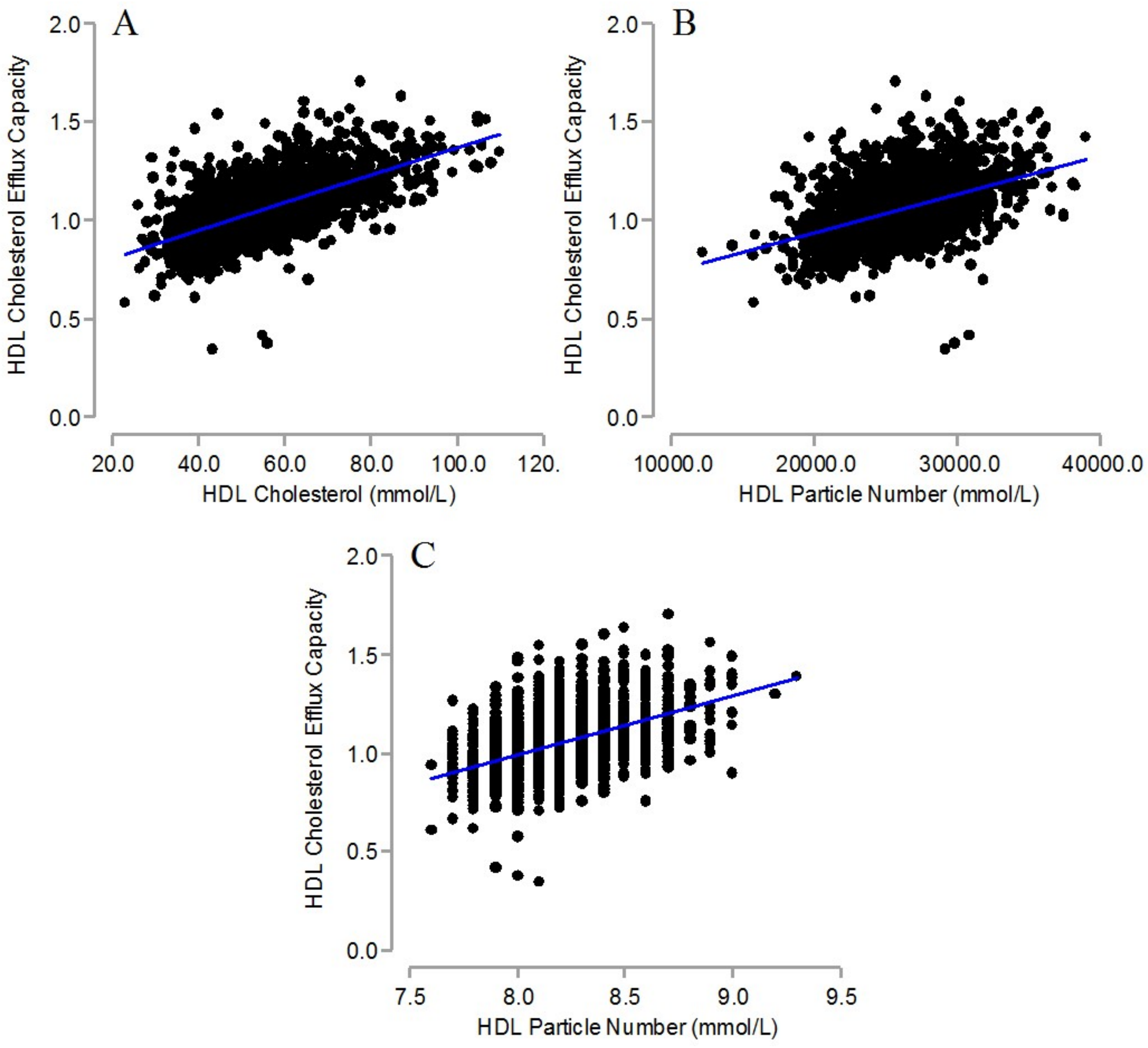

Scatterplots of the relationships of CEC with (A) plasma HDL-C, (B) HDL particle number and (C) HDL particle size.

Supplementary Table I: Characteristics of groups selected on basis of relative CEC and plasma HDL-C levels

Variable	Low efflux/High HDL cholesterol (n=35) Mean ± SD*	High efflux/High HDL cholesterol (n=319) Mean ± SD*	High efflux/Low HDL cholesterol (n=42) Mean ± SD*	Low efflux/Low HDL cholesterol (n=316) Mean ± SD*
Age (years)	36.9 ± 14.0	41.1 ± 14.0	41.8 ± 15.5	37.3 ± 14.7
Sex (% Male)	25.7	26.6	71.4	69.0
BMI (kg/m^2)	24.2 ± 4.0	24.7 ± 3.7	28.0 ± 4.4	27.4 ± 5.2
Waist: hip Ratio	0.80 ± 0.08	0.81 ± 0.07	0.91 ± 0.08	0.87 ± 0.08
SBP (mm Hg)	123.6 ± 15.7	128.2 ± 19.6	139.1 ± 24.6	128.1 ± 18.2
DBP (mm Hg)	77.8 ± 10.4	80.6 ± 11.3	86.6 ± 15.4	78.8 ± 11.1
Ever Smoked (%)	65.7	60.2	71.4	59.2
Current Smoker (%)	88.6	84.3	88.1	79.1
Pack Years*	5.5 (2.3-13.5)	4.4 (0.9-15.5)	18.0 (8.4-30.0)	7.5 (3.0-19.5)
Alcohol consumption (units)*	7.0 (1.0-13.0)	9.0 (3.5-17.0)	9.5 (0.3-18.0)	6.0 (0.0-14.0)
Diabetes (%)	0.0	1.6	2.4	1.9
Albumin (g/L)	44.7 ± 2.9	45.3 ± 2.7	44.3 ± 2.8	45.1 ± 2.9
Total cholesterol (mg/dL)	177.9 ± 36.1	199.1 ± 38.2	180.5 ± 41.7	165.0 ± 34.3
Cholesterol ester (mg/dL)	135.0 ± 28.5	149.6 ± 28.8	139.4 ± 32.3	125.2 ± 25.9
Free cholesterol (mg/dL)	42.9 ± 10.3	49.5 ± 10.8	41.2 ± 10.6	39.7 ± 9.6
LDL cholesterol (mg/dL)	100.6 ± 27.2	113.4 ± 29.9	121.5 ± 31.5	109.3 ± 27.8
Triglycerides (mg/dL)*	80.0 (63.5-109.5)	108.0 (78.0-145.0)	206.0 (134.3-317.8)	141.5 (102.8-202.3)
Phospholipids (mg/dL)	224.6 ± 33.6	260.4 ± 40.0	232.5 ± 42.4	200.8 ± 31.2
Free fatty acids (mg/dL)	18.6 ± 8.5	22.5 ± 10.1	23.7 ± 9.9	20.0 ± 8.2
Lipoprotein (a) (mg/dL)*	17.0 (9.0-26.5)	16.0 (7.0-28.0)	21.0 (11.0-25.8)	15.0 (6.8-27.3)
HDL-C (mg/dL)	64.5 ± 4.9	73.5 ± 11.0	42.8 ± 3.9	41.0 ± 4.5
HDL-CE (mg/dL)	37.0 ± 8.3	40.2 ± 9.7	25.9 ± 4.8	25.3 ± 5.0
HDL free cholesterol (mg/dL)	8.26 ± 2.44	9.12 ± 2.49	5.33 ± 0.95	5.44 ± 1.10
HDL triglycerides (mg/dL)	16.1 ± 5.2	18.7 ± 5.9	19.5 ± 6.7	17.5 ± 5.84
HDL phospholipids (mg/dL)	112.9 ± 25.6	131.6 ± 25.9	102.6 ± 22.4	94.4 ± 16.8
HDL particle number (nmol/L)	26224.2 ± 3011.1	28085.9 ± 3760.4	25249.3 ± 3527.8	23469.8 ± 2624.8
Mean HDL particle size (nm)	8.31 ± 0.18	8.40 ± 0.23	8.13 ± 0.23	8.00 ± 0.16
Apolipoprotein A-I (mg/dL)	168.0 ± 13.5	189.9 ± 27.5	137.3 ± 13.9	128.0 ± 14.4
Apolipoprotein A-II (mg/dL)	41.7 ± 7.7	46.9 ± 8.3	40.1 ± 7.0	36.2 ± 5.1
CEC†	0.93 ± 0.05	1.24 ± 0.11	1.20 ± 0.09	0.88 ± 0.07

BMI, body mass index; DBP, diastolic blood pressure; HDL, high-density lipoprotein; LDL, low-density lipoprotein; SBP, systolic blood pressure. Categorical variables are presented as percentages and continuous variables as means and standard deviations. *Medians and interquartile ranges are given instead of means and standard deviations for variables with skewed distributions. †CEC is expressed as a percentage of efflux in the sample, normalized to a reference sample.

Supplementary Table II: Full results of analysis of variables that distinguish individuals with either a low CEC and high HDL-C level or a high CEC with a low HDL-C level.

	Low CEC/High HDL-C Odds Ratio (95% CI)	*P*-value	High CEC/Low HDL-C Odds Ratio (95% CI)	*P*-value
Age	0.89 (0.61,1.29)	0.530	1.38 (1.00,1.92)	0.051
Sex (Female)	1.68 (0.73,3.89)	0.226	0.87 (0.43,1.79)	0.712
BMI	0.78 (0.49,1.27)	0.322	1.19 (0.89,1.60)	0.234
Waist: hip Ratio	0.75 (0.48,1.16)	0.190	1.61 (1.13,2.29)	0.008
SBP	0.79 (0.53,1.18)	0.247	1.71 (1.27,2.31)	$4.09x10^{-4}$
DBP	0.74 (0.50,1.12)	0.158	1.84 (1.35,2.50)	$9.98x10^{-5}$
Ever Smoked (Yes)	0.65 (0.30,1.40)	0.273	0.59 (0.29,1.20)	0.146
Current Smoker (Yes)	0.49 (0.16,1.50)	0.214	0.48 (0.18,1.27)	0.139
Pack Years	0.77 (0.43, 1.41)	0.401	1.10 (0.83,1.45)	0.505
Alcohol consumption	0.57 (0.34,0.95)	0.031	1.19 (0.85,1.66)	0.304
Albumin	0.69 (0.45,1.04)	0.076	0.72 (0.51,1.00)	0.053
Total cholesterol	0.71 (0.48,1.06)	0.096	1.47 (1.04,2.07)	0.028
Cholesterol ester	0.74 (0.50,1.09)	0.131	1.59 (1.14,2.23)	0.007
Free cholesterol	0.68 (0.44,1.02)	0.064	1.08 (0.75,1.55)	0.679
LDL cholesterol	0.65 (0.44,0.96)	0.029	1.49 (1.06,2.09)	0.02
Triglycerides*	0.20 (0.08,0.51)	$7.17x10^{-4}$	2.03 (1.55,2.65)	$2.35x10^{-7}$
Phospholipids	0.54 (0.36,0.81)	0.002	2.63 (1.78,3.89)	$1.20x10^{-6}$
Free fatty acids	0.62 (0.41,0.95)	0.026	1.53 (1.12,2.09)	0.008
Lipoprotein (a)	0.92 (0.61,1.39)	0.697	1.17 (0.84,1.63)	0.35
HDL-C ester	1.07 (0.69,1.67)	0.759	0.86 (0.47,1.59)	0.63
HDL free cholesterol	1.13 (0.73,1.74)	0.597	0.52 (0.25,1.07)	0.074
HDL triglycerides	0.61 (0.40,0.93)	0.022	1.45 (1.07,1.98)	0.018
HDL phospholipids	0.59 (0.40,0.89)	0.012	1.88 (1.13,3.14)	0.015
HDL particle number	0.68 (0.45,1.01)	0.055	2.01 (1.32,3.05)	0.001
Mean HDL particle size	1.00 (0.59,1.69)	0.987	2.82 (1.75,4.54)	$2.01x10^{-5}$
Apolipoprotein A-I	0.67 (0.40,1.11)	0.12	3.56 (1.62,7.81)	0.002
Apolipoprotein A-II	0.60 (0.41,0.86)	0.006	2.45 (1.52,3.94)	$2.45x10^{-4}$

Odds of having high versus low CEC given high HDL-C level (left) or low versus high CEC given low HDL-C level (right). Odds ratios represent change in risk for a 1 SD change in each variable except for categorical variables. Results relate to univariate analyses but include an adjustment for HDL-C as a covariate, due to differences in the distribution of HDL-C between groups. Note that diabetes was not included due to low numbers. *Reaches a Bonferroni level of significance of 0.002 (accounting 27 tests).*

Supplementary Table III: Associations with HDL-C, CEC and CEC adjusted for HDL-C for selected variants associated with HDL-C in genome-wide association studies

Locus	SNP	CHR	POS	EA	AF	INFO	HDL-C Beta (95% CI)	HDL-C Pvalue	CEC Beta (95% CI)	CEC Pvalue	CEC HDL-C Adjusted Beta (95% CI)	CEC HDL-C Adjusted Pvalue
**PIGV-NR0B2**	rs12748152	1	27,138,393	T	0.094	1.00	-0.07 (-0.21 , 0.06)	0.274	-0.15 (-0.30 , 0.01)	0.059	-0.07 (-0.19 , 0.05)	0.282
**PABPC4**	rs4660293	1	40,028,180	G	0.245	0.99	-0.05 (-0.14 , 0.04)	0.300	-0.03 (-0.14 , 0.08)	0.619	-0.01 (-0.09 , 0.08)	0.859
**HDGF-PMVK**	rs12145743	1	156,700,651	G	0.333	1.00	0.05 (-0.03 , 0.14)	0.216	0.03 (-0.07 , 0.13)	0.546	-0.02 (-0.10 , 0.05)	0.574
**ANGPTL1**	rs4650994	1	178,515,312	A	0.464	1.00	0.02 (-0.06 , 0.10)	0.583	0.02 (-0.07 , 0.11)	0.643	-0.01 (-0.08 , 0.06)	0.886
**ZNF648**	rs1689800	1	182,168,885	G	0.339	0.99	-0.06 (-0.15 , 0.02)	0.154	-0.08 (-0.18 , 0.02)	0.105	-0.05 (-0.12 , 0.03)	0.230
**GALNT2**	rs4846914	1	230,295,691	A	0.386	0.98	-0.02 (-0.10 , 0.06)	0.624	-0.03 (-0.12 , 0.07)	0.578	-0.02 (-0.09 , 0.06)	0.684
**COBLL1**	rs12328675	2	165,540,800	C	0.123	1.00	0.09 (-0.03 , 0.21)	0.149	0.13 (-0.00 , 0.27)	0.057	0.04 (-0.07 , 0.15)	0.457
**CPS1**	rs1047891	2	211,540,507	A	0.345	0.92	-0.07 (-0.16 , 0.02)	0.134	-0.07 (-0.17 , 0.04)	0.206	0.00 (-0.08 , 0.08)	0.966
**IRS1**	rs2972146	2	227,100,698	T	0.355	1.00	0.01 (-0.08 , 0.09)	0.858	-0.04 (-0.14 , 0.05)	0.388	-0.05 (-0.13 , 0.02)	0.168
**ATG7**	rs2606736	3	11,400,249	T	0.373	1.00	0.05 (-0.03 , 0.13)	0.233	0.08 (-0.01 , 0.18)	0.072	0.03 (-0.04 , 0.11)	0.348
**SETD2**	rs2290547	3	47,061,183	A	0.173	0.93	-0.09 (-0.20 , 0.02)	0.107	-0.09 (-0.21 , 0.03)	0.152	-0.04 (-0.13 , 0.06)	0.475
**RBM5**	rs2013208	3	50,129,399	T	0.473	1.00	-0.01 (-0.09 , 0.07)	0.771	-0.02 (-0.11 , 0.07)	0.619	-0.04 (-0.11 , 0.03)	0.280
**STAB1**	rs13326165	3	52,532,118	G	0.212	0.94	0.09 (-0.01 , 0.19)	0.064	0.12 (0.00 , 0.23)	0.046	0.06 (-0.03 , 0.15)	0.190
**GSK3B**	rs6805251	3	119,560,606	C	0.405	1.00	-0.00 (-0.08 , 0.08)	0.956	0.02 (-0.08 , 0.11)	0.723	0.03 (-0.04 , 0.10)	0.415
**C4orf52**	rs10019888	4	26,062,990	G	0.173	0.99	-0.11 (-0.21 , -0.00)	0.045	0.00 (-0.11 , 0.12)	0.946	0.07 (-0.02 , 0.16)	0.136
**FAM13A**	rs3822072	4	89,741,269	A	0.448	0.98	-0.01 (-0.09 , 0.07)	0.732	-0.03 (-0.12 , 0.06)	0.564	-0.02 (-0.09 , 0.05)	0.618
**ADH5**	rs2602836	4	100,014,805	G	0.412	1.00	0.02 (-0.06 , 0.10)	0.644	0.02 (-0.08 , 0.11)	0.745	-0.02 (-0.09 , 0.06)	0.624
**SLC39A8**	rs13107325	4	103,188,709	T	0.074	1.00	0.02 (-0.13 , 0.17)	0.764	-0.18 (-0.36 , -0.01)	0.038	-0.12 (-0.26 , 0.01)	0.078
**ARL15**	rs6450176	5	53,298,025	A	0.261	1.00	-0.04 (-0.13 , 0.05)	0.426	0.07 (-0.03 , 0.17)	0.194	0.10 (0.02 , 0.18)	0.019
**RSPO3**	rs1936800	6	127,436,064	T	0.476	0.98	0.05 (-0.03 , 0.13)	0.195	0.01 (-0.08 , 0.10)	0.831	-0.01 (-0.08 , 0.06)	0.764
**CITED2**	rs605066	6	139,829,666	T	0.433	0.99	-0.07 (-0.15 , 0.01)	0.092	-0.12 (-0.21 , -0.02)	0.014	-0.07 (-0.15 , -0.00)	0.041
**DAGLB**	rs702485	7	6,449,272	G	0.459	1.00	-0.02 (-0.10 , 0.05)	0.550	0.06 (-0.03 , 0.15)	0.220	0.04 (-0.03 , 0.11)	0.310
**SNX13**	rs4142995	7	17,919,258	T	0.411	1.00	-0.06 (-0.14 , 0.02)	0.168	0.02 (-0.07 , 0.12)	0.650	0.06 (-0.01 , 0.14)	0.096

**IKZF1**	rs4917014	7	50,305,863	G	0.311	1.00	0.04 (-0.05 , 0.13)	0.365	0.06 (-0.04 , 0.16)	0.241	0.04 (-0.04 , 0.11)	0.370
**KLF14**	rs4731702	7	130,433,384	T	0.499	0.99	-0.00 (-0.08 , 0.08)	0.978	-0.01 (-0.10 , 0.08)	0.805	-0.01 (-0.08 , 0.06)	0.745
**TMEM176A**	rs17173637	7	150,529,449	C	0.083	1.00	0.08 (-0.06 , 0.22)	0.261	0.03 (-0.13 , 0.19)	0.733	-0.01 (-0.14 , 0.11)	0.830
**PPP1R3B**	rs9987289	8	9,183,358	G	0.099	0.99	-0.02 (-0.15 , 0.11)	0.754	0.03 (-0.12 , 0.18)	0.699	0.01 (-0.10 , 0.13)	0.850
**TRPS1**	rs2293889	8	116,599,199	G	0.421	0.99	-0.04 (-0.12 , 0.04)	0.277	-0.04 (-0.13 , 0.05)	0.372	-0.01 (-0.09 , 0.06)	0.684
**TTC39B**	rs581080	9	15,305,378	C	0.172	0.96	-0.07 (-0.17 , 0.04)	0.205	0.04 (-0.08 , 0.16)	0.503	0.10 (0.00 , 0.19)	0.045
**ABCA1**	rs1883025	9	107,664,301	T	0.249	1.00	-0.06 (-0.15 , 0.03)	0.188	-0.02 (-0.13 , 0.08)	0.683	0.01 (-0.07 , 0.09)	0.765
**MARCH8-ALOX5**	rs970548	10	46,013,277	C	0.239	1.00	-0.06 (-0.15 , 0.04)	0.224	-0.11 (-0.22 , 0.00)	0.051	-0.09 (-0.17 , -0.00)	0.038
**AMPD3**	rs2923084	11	10,388,782	G	0.193	1.00	-0.06 (-0.16 , 0.04)	0.257	0.05 (-0.06 , 0.17)	0.375	0.09 (-0.00 , 0.18)	0.054
**LRP4**	rs3136441	11	46,743,247	C	0.131	1.00	-0.06 (-0.17 , 0.06)	0.310	-0.02 (-0.16 , 0.11)	0.734	0.01 (-0.10 , 0.11)	0.885
**OR4C46**	rs11246602	11	51,512,090	C	0.127	1.00	-0.12 (-0.24 , -0.00)	0.042	-0.11 (-0.25 , 0.03)	0.126	-0.02 (-0.12 , 0.09)	0.770
**KAT5**	rs12801636	11	65,391,317	A	0.225	1.00	0.05 (-0.05 , 0.15)	0.306	0.08 (-0.03 , 0.19)	0.172	0.04 (-0.04 , 0.13)	0.329
**MOGAT2-DGAT2**	rs499974	11	75,455,021	A	0.167	1.00	0.04 (-0.06 , 0.15)	0.431	-0.04 (-0.16 , 0.08)	0.506	-0.05 (-0.15 , 0.04)	0.250
**PDE3A**	rs7134375	12	20,473,758	A	0.431	1.00	-0.01 (-0.09 , 0.07)	0.812	-0.02 (-0.11 , 0.07)	0.705	-0.00 (-0.08 , 0.07)	0.924
**MVK**	rs7134594	12	110,000,193	T	0.462	1.00	0.00 (-0.08 , 0.08)	0.922	0.01 (-0.08 , 0.10)	0.873	0.01 (-0.06 , 0.09)	0.690
**SBNO1**	rs4759375	12	123,796,238	T	0.111	0.94	-0.02 (-0.15 , 0.11)	0.767	-0.06 (-0.21 , 0.09)	0.402	-0.04 (-0.16 , 0.07)	0.461
**ZNF664**	rs4765127	12	124,460,167	T	0.328	0.99	0.11 (0.03 , 0.20)	0.011	0.04 (-0.06 , 0.14)	0.427	-0.01 (-0.09 , 0.07)	0.743
**SCARB1**	rs838880	12	125,261,593	T	0.314	0.94	-0.00 (-0.09 , 0.08)	0.949	-0.00 (-0.10 , 0.10)	0.952	0.01 (-0.07 , 0.09)	0.853
**ZBTB42-AKT1**	rs4983559	14	105,277,209	A	0.372	1.00	-0.05 (-0.13 , 0.03)	0.213	-0.07 (-0.16 , 0.02)	0.133	-0.01 (-0.09 , 0.06)	0.718
**LIPC**	rs1532085	15	58,683,366	G	0.380	1.00	0.14 (0.06 , 0.22)	6.00E-04	0.13 (0.03 , 0.22)	7.70E-03	0.05 (-0.02 , 0.12)	0.183
**LACTB**	rs2652834	15	63,396,867	G	0.174	1.00	0.00 (-0.11 , 0.11)	0.983	-0.03 (-0.15 , 0.09)	0.65	-0.06 (-0.16 , 0.03)	0.198
**FTO**	rs1121980	16	53,809,247	A	0.438	1.00	0.04 (-0.05 , 0.12)	0.386	0.07 (-0.03 , 0.16)	0.161	0.04 (-0.03 , 0.11)	0.269
**CETP**	rs3764261	16	56,993,324	A	0.321	1.00	0.24 (0.15 , 0.32)	2.30E-08	0.09 (0.00 , 0.19)	0.048	-0.07 (-0.15 , 0.00)	0.050
**LCAT**	rs16942887	16	67,928,042	A	0.122	1.00	0.07 (-0.05 , 0.20)	0.257	0.04 (-0.10 , 0.19)	0.54	-0.00 (-0.11 , 0.11)	0.992
**CMIP**	rs2925979	16	81,534,790	C	0.309	1.00	-0.06 (-0.14 , 0.02)	0.151	-0.00 (-0.10 , 0.09)	0.96	0.04 (-0.03 , 0.11)	0.266
**STARD3**	rs11869286	17	37,813,856	C	0.327	1.00	-0.08 (-0.16 , 0.01)	0.084	-0.03 (-0.13 , 0.07)	0.545	0.02 (-0.06 , 0.09)	0.693
**ABCA8**	rs4148008	17	66,875,294	G	0.299	0.99	-0.03 (-0.11 , 0.05)	0.471	-0.06 (-0.15 , 0.04)	0.229	-0.01 (-0.08 , 0.07)	0.814
**PGS1**	rs4129767	17	76,403,984	A	0.496	1.00	-0.07 (-0.14 , 0.01)	0.102	-0.04 (-0.13 , 0.05)	0.343	-0.02 (-0.09 , 0.05)	0.617
**LIPG**	rs7241918	18	47,160,953	T	0.186	1.00	-0.14 (-0.25 , -0.04)	6.80E-03	-0.07 (-0.19 , 0.05)	0.267	0.03 (-0.06 , 0.12)	0.548

**MC4R**	rs12967135	18	57,849,023	A	0.259	1.00	-0.04 (-0.12 , 0.05)	0.436	-0.03 (-0.14 , 0.07)	0.525	-0.02 (-0.10 , 0.06)	0.560
**ANGPTL4**	rs7255436	19	8,433,196	A	0.485	1.00	0.01 (-0.07 , 0.09)	0.775	0.10 (0.01 , 0.19)	0.036	0.04 (-0.03 , 0.11)	0.274
**ANGPTL8**	rs737337	19	11,347,493	C	0.074	1.00	-0.13 (-0.27 , 0.02)	0.088	-0.25 (-0.43 , -0.08)	4.40E-03	-0.13 (-0.27 , 0.00)	0.053
**HAS1**	rs17695224	19	52,324,216	A	0.246	1.00	0.01 (-0.08 , 0.11)	0.775	-0.05 (-0.16 , 0.06)	0.356	-0.06 (-0.14 , 0.03)	0.184
**LILRA3**	rs386000	19	54,792,761	C	0.245	0.95	0.04 (-0.06 , 0.13)	0.439	0.01 (-0.10 , 0.11)	0.917	-0.04 (-0.12 , 0.05)	0.385
**HNF4A**	rs1800961	20	43,042,364	T	0.031	1.00	0.02 (-0.21 , 0.26)	0.841	-0.09 (-0.35 , 0.18)	0.529	-0.08 (-0.29 , 0.13)	0.453
**PLTP**	rs6065906	20	44,554,015	C	0.195	1.00	-0.05 (-0.15 , 0.05)	0.363	-0.04 (-0.15 , 0.08)	0.523	0.02 (-0.07 , 0.11)	0.670
**UBE2L3**	rs181362	22	21,932,068	T	0.196	1.00	-0.12 (-0.23 , -0.02)	0.021	-0.06 (-0.18 , 0.06)	0.330	0.02 (-0.07 , 0.12)	0.623

SNP, lead single nucleotide variant at locus associated with HDL-C in genome-wide study reported by the Global Lipids Genetics Consortium (Ref 19 in main paper); CHR, chromosome; POS, position on chromosome in base-pairs; EA; effect (minor) allele; AF, minor allele frequency; INFO, quality of imputation in GRAPHIC; Betas are reported for the minor allele. HDL-C analyses were adjusted for age and sex while both CEC analyses were additionally adjusted for CEC plate and CEC batch.
