# Supplementary figures and images for: Large-Scale Analysis of Determinants, Stability, and Heritability of High-Density Lipoprotein Cholesterol Efflux Capacity
Source: Arterioscler Thromb Vasc Biol. 2017 Sep 27;37(10):1956–62. doi: 10.1161/ATVBAHA.117.309201 (PMC5627541; doi:10.1161/ATVBAHA.117.309201)

# Determinants of HDL-efflux

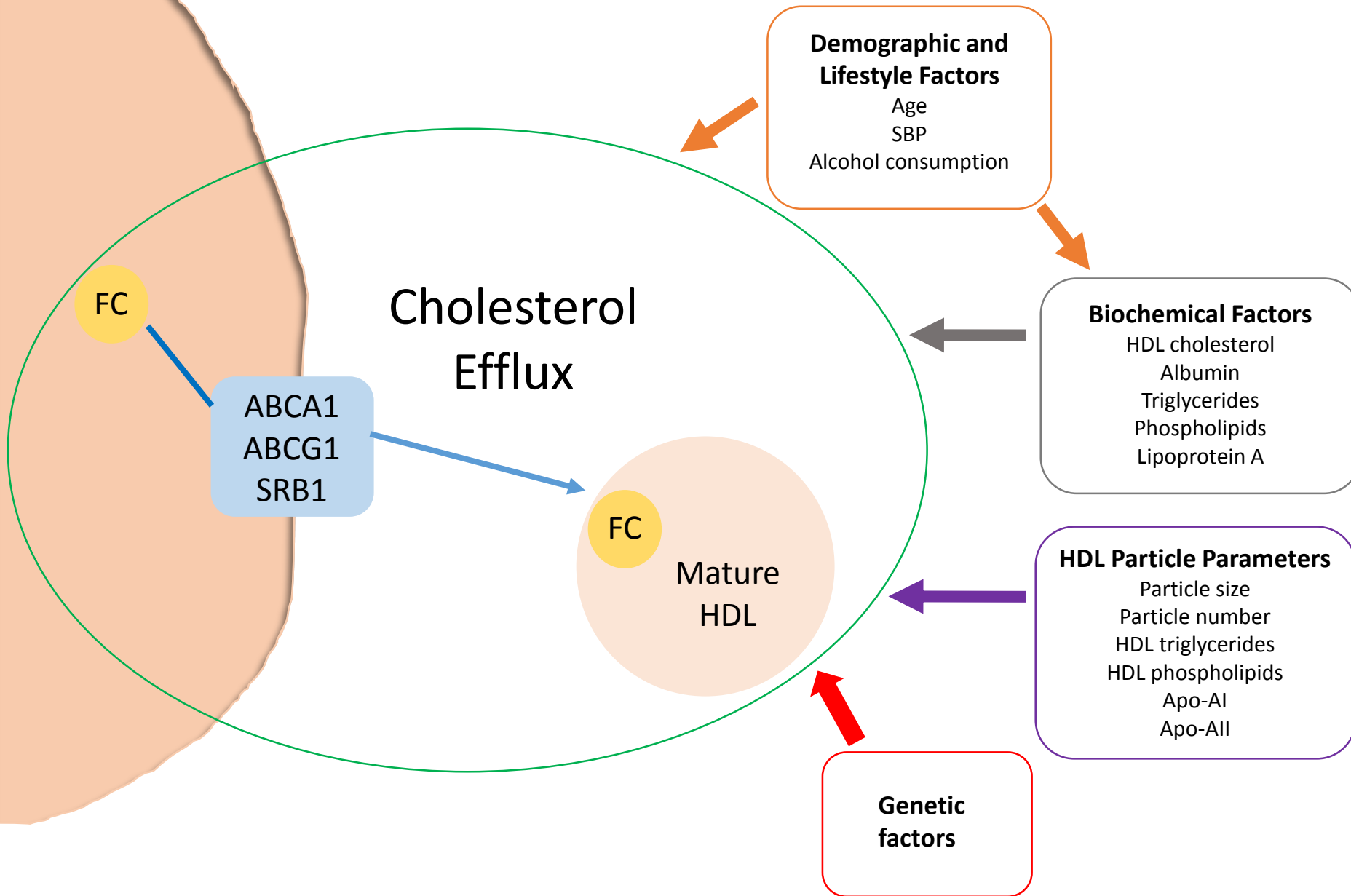

Supplement: Supplementary file 3 [file atv-37-1956-s003.pdf]
